# Management of Complex Root Canal Curvature of Bilateral Radix Entomolaris: Three-Dimensional Analysis with Cone Beam Computed Tomography

**DOI:** 10.1155/2013/697323

**Published:** 2013-12-21

**Authors:** Muktishree Mahendra, Anand Verma, Sanjeev Tyagi, Santosh Singh, Kapil Malviya, Ramit Chaddha

**Affiliations:** ^1^Department of Conservative Dentistry & Endodontics, People's Dental Academy, Bhopal, Madhya Pradesh 462037, India; ^2^Anand Verma Dental Clinic, C/2-30, Civil Lines, Shamla Hill, Bhopal 462002, India

## Abstract

The meticulous knowledge of anatomic characteristics and their variations is essential for the clinician. Radix entomolaris (RE) is one such anomaly where an extra root is present on the distolingual aspect of the mandibular first molar. 18-year-old patient was referred for the root canal treatment of mandibular right and left first molars. Intraoral periapical radiograph revealed additional periodontal spacing crossing distal root of 36. A CBCT was advised and it confirmed the presence of extra roots both in 36 and 46. CBCT is useful in endodontics as it aids in the identification of essential anatomic structures and determination of radius and angle of root canal curvature which is linked to fracture of the instrument. The classical triangular access cavity was modified to a trapezoidal form to locate the extra canal. All canals were instrumented with controlled memory nickel titanium instruments and obturation was done with single cone technique.

## 1. Introduction

Success of endodontic therapy depends on the complete cleaning and shaping of the root canal systems, tridimensional obturation, and proper coronary sealing. For that purpose, the knowledge of the morphology of the root canal system as well as its variations is essential. The mandibular first molar is known to display several anatomical variations. The number of roots may also vary in mandibular molars, in which a third additional root, firstly mentioned in the literature by Carabelli [1], is called radix entomolaris. This supernumerary root is located in distolingual position, mainly in the mandibular first molars. When located in the mesiobuccal surface, the anomaly is called radix paramolaris [2]. The frequency of radix entomolaris is less than 5% in Caucasian, African, Eurasian, and Indian populations [3], whereas its bilateral occurrence is less than 2.19% [4]. This extra root is typically smaller and more sharply curved than the distobuccal (DB) root, requiring special attention when root canal treatment is being considered.

CBCT is a noninvasive technique to determine the occurrence of distolingual root and reveals the true nature of macrostructure three-dimensionally and its curvature and angulation [5]. CBCT shows the exact position of distolingual root and hence it helps in tracking the curvature and prevents iatrogenic event that might occur in relation to canal curvature like instrument separation, perforation, ledge formation, and so forth.

## 2. Case Presentation

18-year-old male patient reported to the dental office with the chief complaint of swelling in lower left back region for last two days as well as pain in lower right back region. The pain was aggravated on taking cold and hot food items and on mastication. The clinical examination revealed the carious right and left mandibular first molars. His medical history was noncontributory.

**Figure 1:**
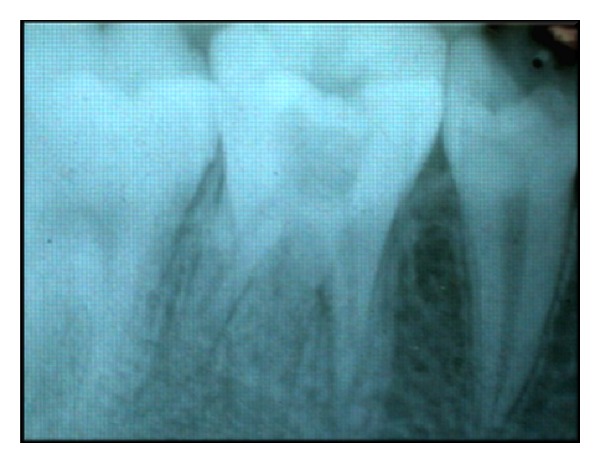
Preoperative IOPA of 36.

**Figure 2:**
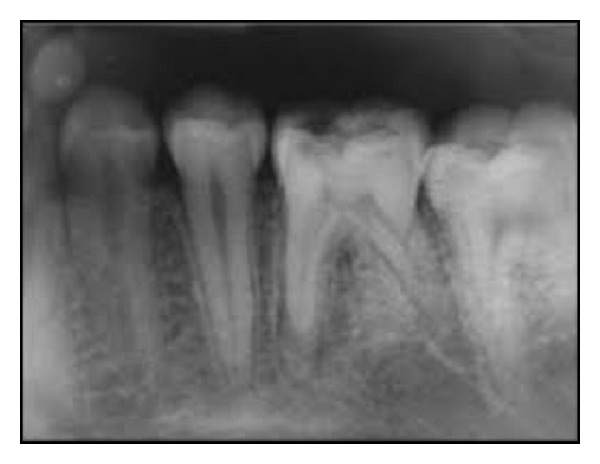
Preoperative IOPA of 46.

Intraoral periapical radiograph of 36 (Figure 1) revealed deep carious lesion involving the pulp with periapical radiolucency at the root apices and presence of an extra root in 36. On close inspection of radiograph of 46 (Figure 2), an impression of double periodontal ligament space on distal side leads to the suspicion of additional root entity with 46. Based on clinical and radiographic examination, diagnosis of acute apical abscess with 36 and symptomatic irreversible pulpitis with acute apical periodontitis of 46 was established.

**Figure 3:**
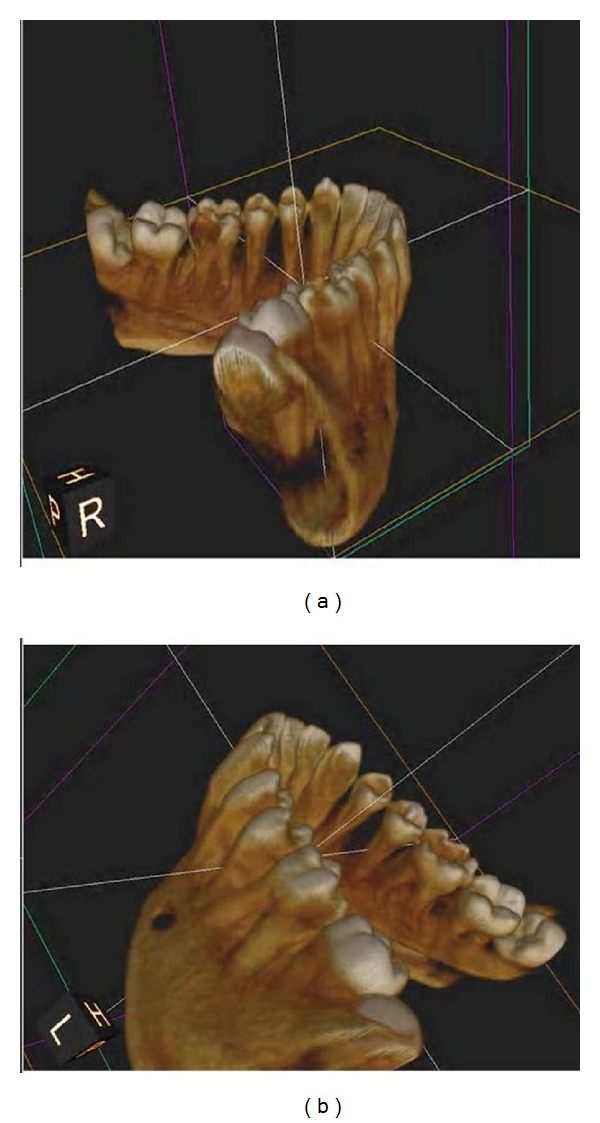
Oblique slicing showing root canal curvature of distolingual root of 36 and 46.

**Figure 4:**
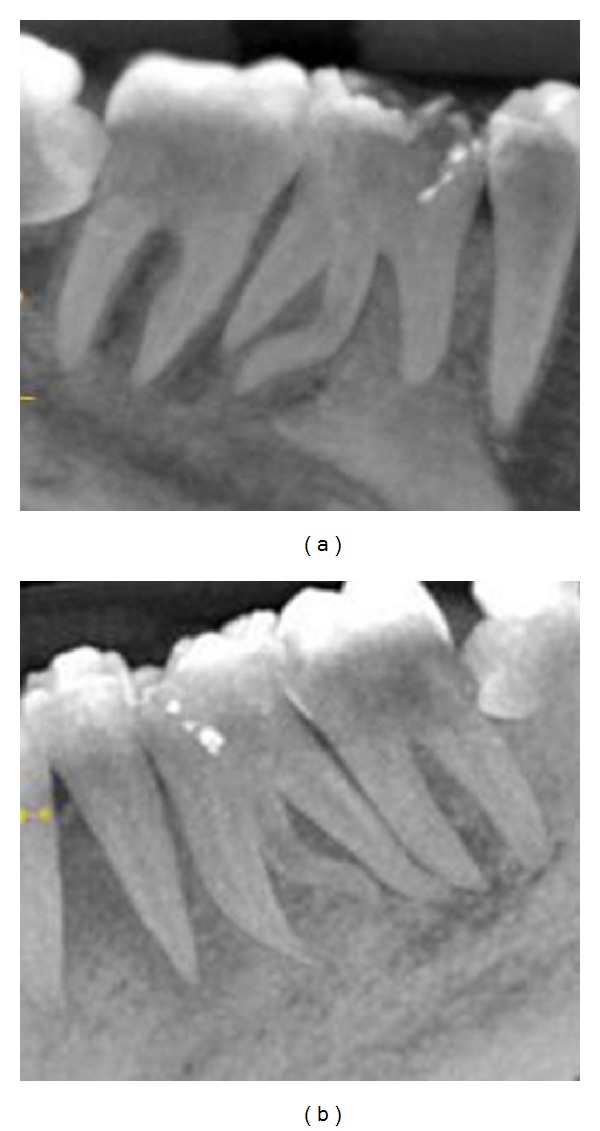
Sagittal view of 36 and 46 showing root canal curvature of distolingual root.

**Figure 5:**
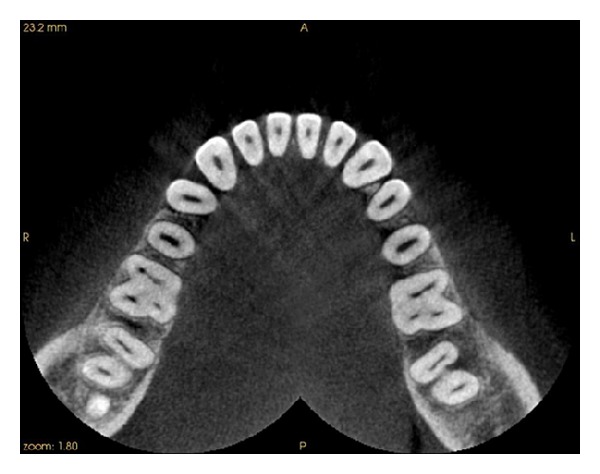
Axial views allow the relationship between the additional root and other roots to be assessed.

**Figure 6:**
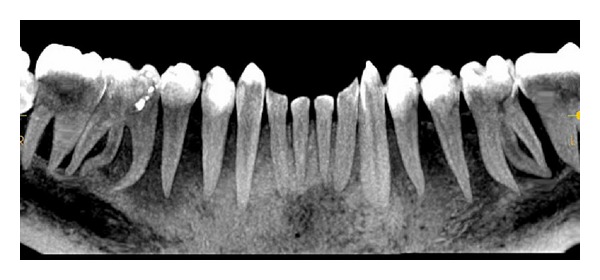
Curved slicing section showing 36 and 46.

**Figure 7:**
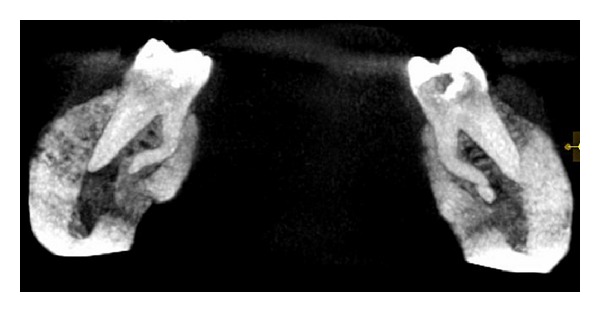
Coronal section showing 36 and 46.

A cone beam computerized tomography was advised to confirm the diagnosis of distolingual root. CBCT was done and determined the exact position, angle, and curvature of distolingual root of the permanent mandibular first molars. The morphology of teeth was obtained in oblique (Figure 3), sagittal (Figure 4), axial (Figure 5), curve slicing (Figure 6), and coronal section (Figure 7).

After adequate anaesthesia and isolation with rubber dam, access cavity was prepared using endoaccess kit in the mandibular left first molar. The cavity design was slightly modified by extending more distolingually using ultrasonic tips for exploring extra distal canal orifice. CBCT imaging had shown the severe curvature from the middle third to the apical third in the distolingual root; therefore, the canals were negotiated with pathfinder files. These files create glide path with .02 rotary Ni-Ti instruments till the working length and they glide through the canal and pasts the curvature smoothly. Instrumentation was completed with .04 tapered number 35 Ni-Ti rotary instruments in a crown down fashion till the working length. All the canals were irrigated with 2.5% sodium hypochlorite and 17% EDTA. Calcium Hydroxide was placed into the canals as intracanal medicament. After two weeks, the tooth was asymptomatic and final irrigation was done with ultrasonic passive irrigation for 1 min. Canals were dried with paper point and obturation was done using gutta-percha points and endoflas sealer. Later, the access cavity was sealed with composite.

**Figure 8:**
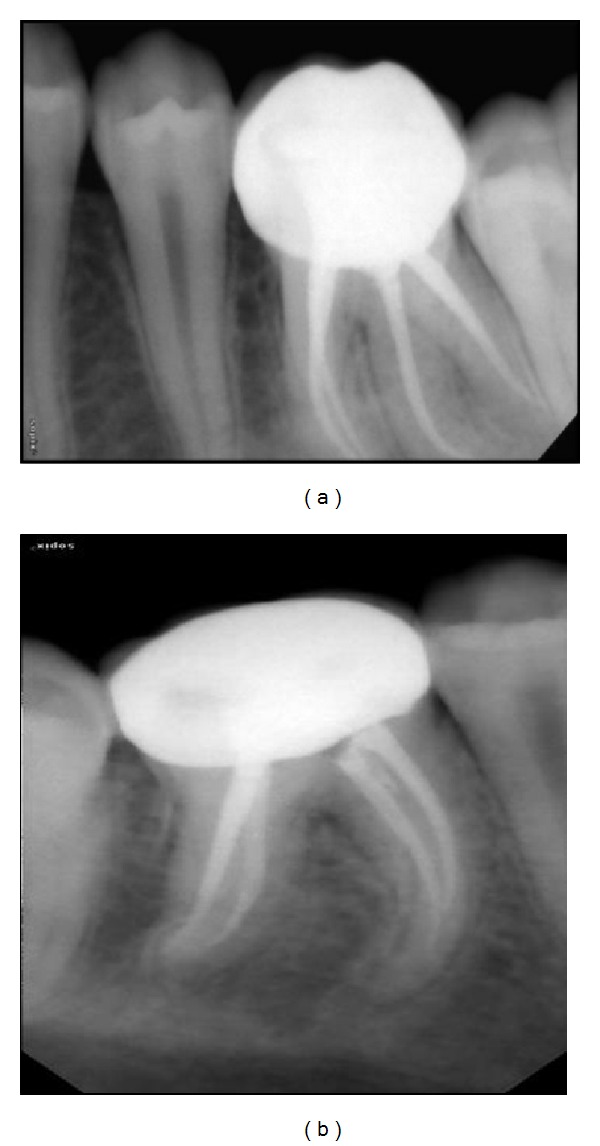
Postoperative IOPA of 36 and 46.

After completion of 36, endodontic treatment of the mandibular right first molar was initiated. After creating the glide path, biomechanical preparation was using controlled memory NiTi instruments. The canals were irrigated with 2.5% NaOCl and normal saline. Calcium hydroxide was used as intracanal medicament and access opening was sealed with Zinc oxide-eugenol cement. One week later, the tooth was asymptomatic, the canals were dried with paper point, obturation was completed, and access cavity was sealed with composite. Patient was recalled after 1 month and tooth preparation for porcelain fused to metal crown (Figure 8) was done and cemented subsequently.

## 3. Discussion

The success of endodontic therapy depends on the root canal morphology to some extent, so the clinician should have a thorough understanding of normal anatomy as well as its unusual anatomical configurations. The root canals of the mandibular permanent first molars have several typical anatomical features and anomalies. The presence of four canals is relatively frequent [6], but the presence of two distal roots is uncommon and bilateral presence of three rooted mandibular molar is a rare case. This additional third root, first mentioned in the literature by Carabelli [1] (1844), is called the radix entomolaris (RE), located distolingually in the mandibular molars, mainly the first molars.

The coronal third of distolingual root of RE is fixed partially and completely to the distal root. Based on buccolingual orientation, De Moor et al. [7] have classified RE into three types. Type I refers to straight root or canal. Type II refers to an initially curved entrance which continues as a straight root/root canal. Type III refers to an initial curve in the coronal third of the root canal and a second curve beginning in the middle and continuing to the apical third. In this case, distolingual root of 36 is classified as Type III because severe canal curvature is present in middle third till the apical end. Distolingual root of RE is mostly in buccolingual plane as in conventional radiography (two-dimensional); tooth is visualized in mesiodistal plane only. CBCT [8] is a three-dimensional imaging overcoming these major limitations by visualization of the third dimension and ascertaining the exact location and anatomy of RE.

The location of distolingual canal orifice has an implication for access cavity preparation. The orifice of RE is located far distally from the main distal canal. Hence, the modification of the classical triangular access cavity to a trapezoidal form is required to locate the additional canal orifice and establish the straight line access. The interorifice distance between extra distolingual canal and remaining canals can be measured with CBCT, serving as a useful guideline to locate and treat RE [5, 8]. The axial sectioning of CBCT allows for the exact visualization of distolingual orifice in relation to other canals. Ultrasonic tips help in careful removal of dentin and thus prevent weakening of tooth structure [9].

The determination of root canal curvature is a key procedure for endodontic planning. Knowledge of root curvature radius allows for selection of NiTi rotary files and accurate planning of root canal instrumentation. NiTi rotary files undergoe hundreds of cycles of alternating compression and flexure when placed in curved canal. Radius of curvature is the most important factor influencing the cyclic fatigue. The more severe the angle of curvature with small radius of curvature, the greater the cyclic fatigue and thus, lower its life expectancy [10]. Instrument separation increased as radius of curvature decreased. Thus, file design features such as NiTi core diameter, cross-sectional shape, and flute depth should be taken into account while selecting the NiTi rotary instrument for curved canals. In this case, new set of controlled memory flexible NiTi files has been used as they have high resistance to cyclic fatigue and reduce the incidence of instrument separation.

## 4. Conclusion

Cone beam computed tomography is a practical, noninvasive tool for morphological analysis. It also reduces the radiation dose to which patients are exposed. It helps in accurate diagnosis of the third root and can avoid complications or a “missed canal” during root canal treatment. Failing to realize curvature and radii of canal before treatment can lead to preparation errors—apical zips, perforations, canal blockages, or instrument separation—which can leave the canal unprepared and compromise the outcome of the treatment. Additional aids that help in location of orifices are following law of symmetry and orifice location, DG 16 probe, microopener, sodium hypochlorite “bubble” test, long shank burs, ultrasonic instruments, cbct, and operating microscope.
